# The Impact of Support and Reduction Temperature on the Catalytic Activity of Bimetallic Nickel-Zirconium Catalysts in the Hydrocracking Reaction of Algal Oil from Spirulina Platensis

**DOI:** 10.3390/molecules29225380

**Published:** 2024-11-15

**Authors:** Lukasz Szkudlarek, Karolina A. Chalupka-Spiewak, Aleksandra Zimon, Michal Binczarski, Waldemar Maniukiewicz, Pawel Mierczynski, Malgorzata Iwona Szynkowska-Jozwik

**Affiliations:** Chemical Department, Institute of General and Ecological Chemistry, Lodz University of Technology, Zeromskiego 116, 90-543 Lodz, Poland; lukasz.szkudlarek@dokt.p.lodz.pl (L.S.); aleksandra.zimon@p.lodz.pl (A.Z.); michal.binczarski@p.lodz.pl (M.B.); waldemar.maniukiewicz@p.lodz.pl (W.M.); pawel.mierczynski@p.lodz.pl (P.M.); malgorzata.szynkowska@p.lodz.pl (M.I.S.-J.)

**Keywords:** zeolites, nickel catalysts, hydrocracking of algal oil, bimetallic nickel-zirconium catalysts

## Abstract

The aim of this work was to investigate the hydrocracking of algae oil derived from Spirulina Platensis species catalyzed with bi-component nickel-zirconia catalysts supported onto different carriers (BEA, ZSM-5 and Al_2_O_3_) in an autoclave at 320 °C for 2 h with a hydrogen pressure of 75 bar. All catalysts were prepared using the wet co-impregnation method and were characterized by H_2_-TPR, XRD, NH_3_-TPD, BET and SEM-EDS. Before reactions, catalysts were calcined at 600 °C for 4 h in a muffle furnace, then reduced with 5%H_2_-95%Ar reducing mixture at 500 °C, 600 °C or 700 °C for 2 h. The obtained products were analyzed and identified by HPLC and GC-MS techniques. In addition to the investigation of the support effect, the influence of the reduction temperature of catalytic systems on the catalytic activity and selectivity of the products was also examined. The activity results show that Ni-Zr systems supported on zeolites exhibited high conversion of algal oil. A gradual decrease in conversion was observed when increasing the reduction temperature of the catalyst (from 500 °C to 600 °C and 700 °C) for BEA zeolite catalysts. The reaction products contain hydrocarbons from C_7_ to C_33_ (for zeolite-supported catalysts) and C_36_ (for systems on Al_2_O_3_). The identified hydrocarbons mainly belong to the gasoil fraction (C_14_–C_22_). In the research, the best catalyst for the algal oil hydrocracking reaction was found to be the 5%Ni-5%Zr/BEA system reduced at 600 °C, which exhibited the second highest algal oil conversion (94.0%). The differences in catalytic activity that occur are due to the differences in the specific surface area among the supports and to differences in the acidity of the catalyst surface depending on the reduction temperature.

## 1. Introduction

Continuous social development over many decades, together with the growth of industry, transport and population, has resulted in an ever-increasing demand for energy [1,2,3,4]. Consumption of fossil fuels to meet these energy needs is associated with the gradual depletion of available deposits. In addition, their combustion has a negative impact on the environment [4,5], due to the emission of harmful gases such as carbon dioxide (CO_2_), carbon monoxide (CO), nitrous oxide and nitrogen oxides (NO_x_), and sulfur oxides (SO_x_) [6]. The non-renewability of the natural resources of oil and natural gas, together with their harmful impact on the environment and the possibility of energy crises due to fuel shortages, are key factors in the search for alternative renewable fuels through, for example, the conversion of vegetable and algal oils [7,8]. The use of animal fats and vegetable and algal oils offers better prospects for obtaining biofuels due to higher conversion rates [4]. Nowadays, third-generation biofuels are being increasingly produced, where conversion processes of algal biomass or algal oils take place. Algae can overcome the drawbacks of production of first- and second-generation biofuels, which makes them a reasonable and promising feedstock [9,10]. The main advantages of using algae are higher oil yields with low breeding and growth requirements and the possibility to grow algae without using agricultural land [11]. Algae are fast-growing organisms that convert sunlight, water and carbon dioxide into biomass by photosynthesis. An additional benefit is that their cultivation can utilize nutrients from municipal and agricultural wastewater, which for traditional harvested plants was harmful and inhibited their growth [12,13]. Also, in comparison with the traditional oleaginous plants, algae are characterized by high sequestration of CO_2_ through high photosynthetic efficiencies, which results in high growth rates [14,15]. As regards the use of algae as a biomass resource for biofuel production, apart from the possibility of hydrocracking oil extracted from algae, they are a potential source of biogas, biodiesel, bioethanol and other value-added products to be obtained on a commercial scale [16].

One of the approaches to produce alternative biofuels is catalytic hydroprocessing, which includes hydrotreating and hydrocracking processes [17]. The main approach to obtaining alternative fuels is hydrocracking. This is a process using bifunctional heterogeneous catalysts capable of converting high molecular weight substrates (such as triglycerides contained in oils) to much lower molecular weight hydrocarbons (when oils are used as substrates, the products are mainly C_15_–C_18_ hydrocarbons) at a high temperature (260–425 °C) in a hydrogen atmosphere under high pressure (35–200 bar). In addition, the products of hydrocracking can be olefinic and aromatic hydrocarbons [18,19]. The production of alkanes through this process is considered an attractive technology for creating hydroprocessed kerosene, green kerosene, hydroprocessed renewable diesel (HRJ), synthetic paraffinic kerosene from hydroprocessed esters and fatty acids (HEFA-SPK), synthetic paraffinic kerosene (SPK) and bio-hydrogenated kerosene (BHK), alternative diesel and jet fuel [20,21]. The hydrocracking process uses catalysts based mostly on precious metals, e.g., platinum (Pt) and systems based on nickel with other elements from group VI (e.g., molybdenum (Mo) or tungsten (W)) [22,23]. Systems based on these elements are well known for their high activity and selectivity in the cracking mechanism. The stability of active metals can be enhanced by dispersing them by introducing them on porous supports [23]. Nickel-based catalysts are becoming increasingly important in thermochemical conversion processes involving hydrogen attachment and oxygen scavenging, due to their high activity at a much lower price compared to noble metals [24].

In catalytic reactions requiring systems with high thermal stability, zeolites are widely used crystalline materials [25]. Zeolites are porous aluminosilicates composed of combined silicate (SiO_4_^−^) and aluminum (AlO_4_^−^) tetrahedra. These materials are mainly used in the chemical industry due to their adsorption and ion exchange capabilities [26,27,28]. In addition, they are also used in catalysis as catalyst carriers due to the uniformity of pores, high surface area, adjustable acid/basic sites (presence of Lewis and Brønsted acid sites) with the ability to modify their acid-base properties and a great variety and availability [26,29,30,31]. Additionally, a beneficial feature of zeolites is shape-selectivity, which supports selectivity of zeolite-based catalytic systems [32].

Industrially, catalysts based on both natural zeolites and those obtained by synthesis, i.e., ZSM-5 (MFI), beta (BEA), zeolites Y (FAU), mordenite (MOR), SAPO-34 (CHA), ZSM-22 (TON) or MCM-22 (MWW), are used [33]. Zeolite ZSM-5, in addition to the previously mentioned advantages along with a higher concentration of acid sites on the surface is characterized by selectivity for aromatic hydrocarbons in reactions, while zeolite BEA features a highly acidic Brønsted nature [34]. All these characteristics make them suitable for use in hydroforming and catalytic cracking processes [34].

Moreover many scientists have conducted research with mesoporous and zeolitic materials enriched with Zr species and observed that the presence of Zr species results in obtaining catalytic materials with special properties [35]. Among the advantages of porous materials with Zr in their structure, the bifunctional character with acidic and hydrogenating properties is mentioned. Despite these special and important properties in hydrocracking processes, such porous materials with Zr species are not tested in this reaction. For this reason, our work was focused on preparation of the catalysts based on BEA and ZSM-5 zeolites and also classical alumina oxide with an addition of Zr species to use them in hydrocracking of algal oil.

Catalysts containing nickel and zirconia were applied in e.g., domino-cyclization or tandem cyclization and hydrogenation of citronellal to menthol (Ni/Zr-Beta) [36,37], hydrogenolysis of glycerol (Ni-Zr/H-beta) [38], catalytic hydrogenation of maleic anhydride to γ-butyrolactone (hierarchical Ni-Zr-MFI) [39], low-temperature CO_2_ methanation (Ni-Zr-Al ternary hydrotalcites) [40].

The objective of this work was to investigate the catalytic activity of bi-component nickel-zirconia catalysts supported onto zeolites (BEA, ZSM-5) and on alumina in the hydrocracking reaction of algal oil. The choice of different supports was to determine their effect on catalyst activity and selectivity. In addition, physico-chemical studies of the obtained systems were carried out using H_2_-TPR, XRD, NH_3_-TPD, BET and SEM-EDS techniques to explain the differences in catalytic activity. The obtained products were analyzed by chromatographic methods (HPLC and GC-MS) to determine composition and selectivity. The results allowed the selection of the most suitable catalyst material for the hydrocracking reaction.

## 2. Results

### 2.1. Textural and Structural Characterization

The BET (Brunauer–Emmet–Teller) surface area with micropore area and external surface area, total pore volume, micropore volume and average pore size of calcined and reduced at various temperatures of bimetallic nickel-zirconium catalysts supported on BEA zeolite, ZSM-5 zeolite and alumina oxide are summarized in Table 1. In the case of Nickel-zirconium BEA zeolite catalysts, the highest specific surface area (SSA, between 445–449 m^2^/g), total pore volume (near 0.63 cm^3^/g) and also the highest average pore radius (between 6.7–7.2 nm) were found. In the case of nickel-zirconium ZSM-5 zeolite catalysts, a higher specific surface area (between 269–277 m^2^/g) and smaller total pore volume (0.06–0.07 cm^3^/g) were observed in comparison with nickel-zirconium supported on alumina oxide catalysts (SSA = 111–115 m^2^/g and total pore volume = 0.21 cm^3^/g), but in both cases the average pore radius was similar and equaled between 2.1–2.6 nm. It should be mentioned that the increase in the temperature reduction does not significantly affect these parameters, which can mean that conditions of the reduction process do not impact on the structure of zeolites and catalytic systems. However, the incorporation of metals on zeolite caused the decrease in the specific surface area of zeolites and their total pore volume, but the average pore radius remained unchanged. Because algal oil consists of long chains of fatty acids, these differences in porosity and structural properties of the obtained bimetallic catalysts can affect their catalytic activity in the hydrocracking of algal oil.

**Figure 1:**
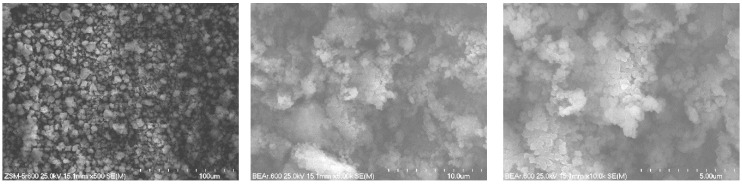
SEM images of 5%Ni-5%ZrBEA catalyst after reduction at 600 °C.

**Figure 2:**
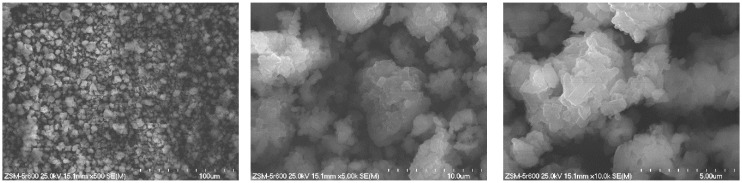
SEM images of 5%Ni-5%ZrZSM-5 catalyst after reduction at 600 °C.

**Figure 3:**
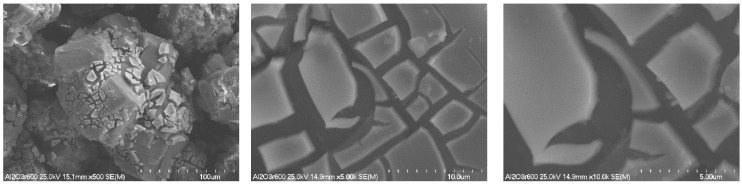
SEM images of 5%Ni-5%Zr/Al_2_O_3_ catalyst after reduction at 600 °C.

The morphology of nickel-zirconium catalysts based on different supports (BEA, ZSM-5 and Al_2_O_3_) after reduction at 600 °C for 2 h in 5%H_2_-95%He gaseous mixture flow was studied by SEM (Scanning Electron Microscopy) method. Figure 1, Figure 2 and Figure 3 present the SEM images obtained for 5%Ni-5%ZrBEA, 5%Ni-5%ZrZSM-5 and 5%Ni-5%Zr/Al_2_O_3_ samples, respectively. Comparing the SEM images obtained for these three catalyst samples, it was found that for zeolite catalysts—5%Ni-5%ZrBEA and 5%Ni-5%ZrZSM-5, the size of observed objects is not higher than 20 µm (Figure 1 and Figure 2). On the surface of BEA zeolite, the smallest oval-shaped objects with the size of 0.2 µm were observed (Figure 1). In the case of the 5%Ni-5%ZrZSM-5 catalyst, objects with a more rectangular shape and the size smaller than 0.4 µm were observed (Figure 2). In Figure 3, the SEM images for 5%Ni-5%Zr/Al_2_O_3_ are shown. In this case, the biggest objects were observed with the size above 100 µm with crashed parts on the surface. This image can indicate the smallest specific surface area of the support and it is in agreement with the BET results.

### 2.2. Reduction Behavior of Prepared Bimetallic Catalytic Systems—TPR and XRD Results

**Figure 4:**
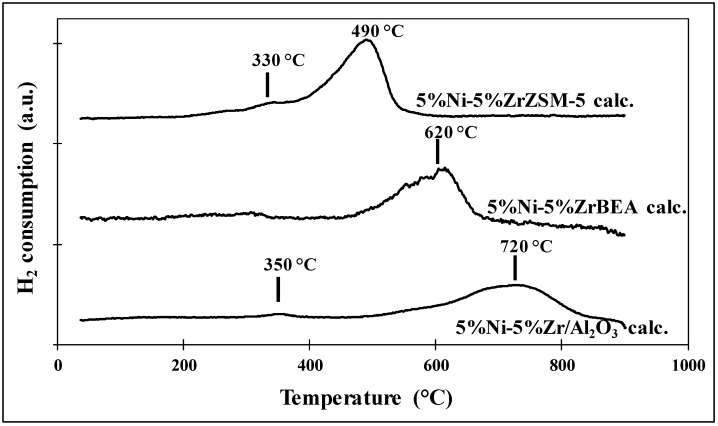
TPR-H_2_ profiles for bimetallic nickel-zirconium catalysts supported on BEA zeolite, ZSM-5 zeolite and Al_2_O_3_ after their calcination at 600 °C for 4 h.

The reducibility of the obtained bimetallic Nickel-Zirconium catalysts was defined by TPR measurements and XRD “in situ”. The TPR-H_2_ profiles performed for 5%Ni-5%ZrBEA zeolite, 5%Ni-5%ZrZSM-5 zeolite and 5%Ni-5%Zr/Al_2_O_3_ are presented in Figure 4. Based on the shapes and the maximum of reduction peaks, it can be said that the character of the reduction process for every catalyst is slightly different, however, for all samples a very small broad peak with the maximum at low temperature between 300–370 °C was observed. This low-temperature peak is probably related to the reduction of nickel oxide in bulk in the case of zeolite catalysts 5%Ni-5%ZrBEA and 5%Ni-5%ZrZSM-5 present on the outer surface of the zeolites and suggests weaker interactions with the zeolite framework, which is in good agreement with the literature data [41,42,43].

In the case of the 5%Ni-5%Zr/A_l2_O_3_ sample, the maximum peak occurs at a slightly higher temperature—350 °C and this is also the region in which nickel oxide reduction weakly interacts with the support.

The second reduction peak with the maximum at higher temperatures could be related to the nickel ions present in the intracrystalline mesopores of zeolites or as isolated ions inside the pores of the zeolite supports and also with the reduction of nickel ions in strong interaction with the supports.

The maximum peak at 490 °C for the 5%Ni-5%ZrZSM-5 zeolite catalyst could be assigned to the reduction of smaller NiO nanoparticles or NiO inside the ZSM-5 pores, or located in ion-exchange positions. A similar effect was also observed by J. F. da Costa-Sierra and co-workers [42], where they concluded that the reduction peak observed at higher temperature is related to the reduction of nickel ions interacting to a higher degree with the zeolite.

**Figure 5:**
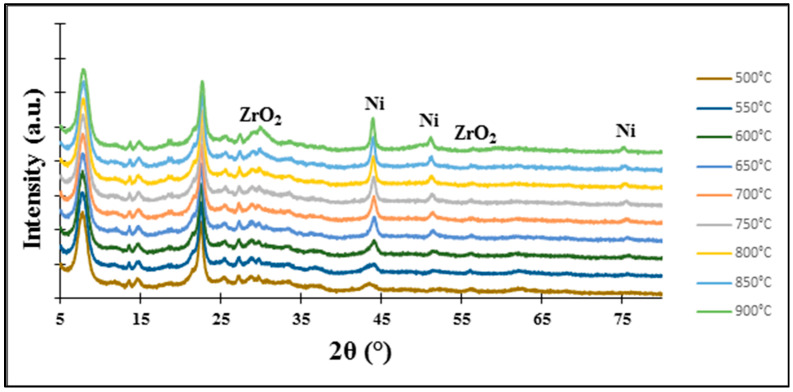
XRD patterns obtained for 5%Ni-5%ZrBEA zeolite during “in situ” measurement in a reduced mixture of 5%H_2_-95%Ar flow in the temperature range 500–900 °C.

**Figure 6:**
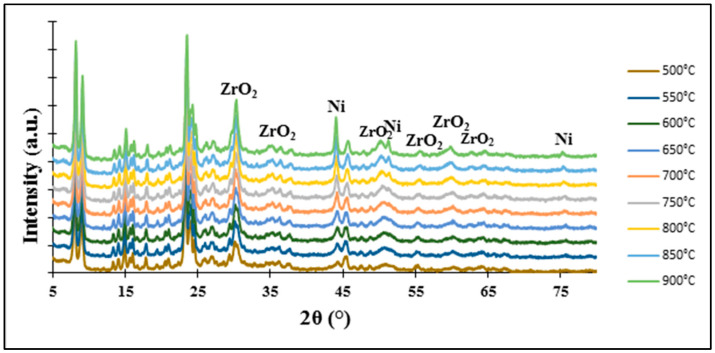
XRD patterns obtained for 5%Ni-5%ZrZSM-5 zeolite during “in situ” measurement in a reduced mixture of 5%H_2_-95%Ar flow in the temperature range 500–900 °C.

In the case of the 5%Ni-5%ZrBEA zeolite sample, the reduction peak with the maximum at 620 °C—a temperature higher in comparison with the 5%Ni-5%ZrZSM-5 zeolite catalyst—was observed. A similar phenomenon was noticed by S. Taghavi et al. and it is probably connected with the size of pores in BEA zeolite and the metal particle dispersion and loading into zeolite. It seems that during impregnation some nickel ions could be ion-exchanged and then stabilized in the zeolite framework. Moreover, some part of NiO formed during calcination could react with bridging protons like Si-O-Al and Ni(OH)+ ions were formed which interact more strongly with the BEA zeolite [43]. The reduction of isolated nickel ions and NiO nanoparticles present in the zeolite pores is more difficult and demands a higher temperature. However, the XRD “in situ” measurements presented in Figure 5 (for 5%Ni-5%ZrBEA zeolite) and Figure 6 (for 5%Ni-5%ZrZSM-5 zeolite) performed for both zeolite catalysts suggest that the temperature of 500 °C is enough for the reduction of nickel ions and nickel oxides present in the extra-framework position and also localized in the pores of zeolites.

**Figure 7:**
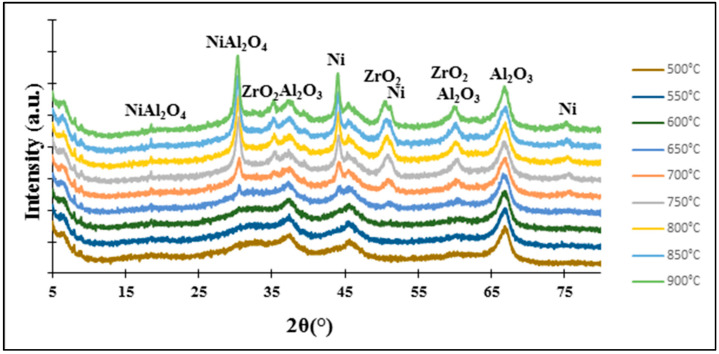
XRD patterns obtained for 5%Ni-5%Zr/Al_2_O_3_ during “in situ” measurement in a reduced mixture of 5%H_2_-95%Ar flow in the temperature range 500–900 °C.

In the case of the 5%Ni-5%Zr/Al_2_O_3_ sample, the second reduction peak was observed in a higher range than in the case of the zeolite catalysts. The maximum peak was found at 720 °C. In connection with the XRD in situ results presented in Figure 7 this high-temperature reduction peak could be assigned to the nickel ions strongly interacting with the alumina oxide and forming spinel NiAl_2_O_4_—the phase which hinders the reduction process.

It should be mentioned that TPR-H_2_ profiles indicate that nickel ions or NiO localized inside the pores of zeolites strongly interact with the support and are the dominant phases for all studied catalysts.

All patterns showed reflections at 2θ = 44.8°, 51.1° and 75.2°, which originate from metallic nickel and according to the research [44,45], these diffraction peaks can be assigned to the (1 1 1), (2 0 0) and (2 2 0) planes of Ni metal, respectively.

The XRD pattern of the 5%Ni-5%Zr/BEA catalyst shows the first two diffraction peaks in all the temperature range, however, the last one was not observed, just for the lowest reduction temperature (500 °C). As regards the 5%Ni-5%Zr/ZSM-5 system, all diffraction peaks from metallic nickel are visible for all reduction temperatures. However, for the 5%Ni-5%Zr/Al_2_O_3_ catalyst sample, the XRD curves show reflections occurring at 2θ angles of 44.8°; and 51.1° from the reduction temperature of 650 °C and above, and the last reflection at a 2θ angle of 75.2° is visible on the pattern from the reduction temperature of 700 °C and higher. Other diffraction peaks visible on the XRD patterns of studied catalytic systems originate from zirconia (ZrO_2_) located at 2θ angles of 28.7°, 30.0°, 34.7°, 35.1°, 50.3°, 56.0°, 59.9° and 62.6°. According to the work written by Lim et al. [44], where Ni@hollow silicate zirconia was synthesized for dry reforming of methane, the authors found reflections at 2θ angles equal to 30.2°, 34.9°, 50.6° and 60.08°, which correspond to the (1 0 1), (0 0 2), (2 0 0), and (2 1 1) reflections of tetragonal zirconia (t-ZrO_2_). Nabgan and co-authors [46] investigated Ni/Co catalytic systems on ZrO_2_ as a support and the X-ray diffraction analysis results in a pattern with reflections at 2θ angle values of 28.19°, 34.45°, 55.62°, 58.3°, 60.02° and 62.83°. The authors attributed these diffractions to the (2 0 0), (2 1 1), (3 2 0), (4 0 0), (4 1 0), (4 1 1), (3 3 1) crystal planes from monoclinic (m-ZrO_2_) and tetragonal zirconia (t-ZrO_2_).

In the case of XRD curves of the 5%Ni-5%Zr/Al_2_O_3_ catalyst subjected to “in situ” reduction, there are also reflections at 2θ angles of 18.6° and 31.0°, which were identified as a NiAl_2_O_4_ spinel phase. This is confirmed by Rajkumar et al. [47], where on the pattern of NiAl_2_O_4_, diffraction peaks located at 2θ = 18.9° and 31.38° were associated with (1 1 1) and (2 2 0) crystal planes of the cubic spinel structure. These reflections were observed in the XRD curves for reduction temperatures above 650 °C for both diffraction peaks.

### 2.3. The Impact of the Reduction Temperature of Bimetallic Ni-Zr Catalysts on Their Acidity—TPD-NH_3_ Results

The temperature-programmed desorption (TPD) of NH_3_ was performed to define the acidity of the catalyst surface. The quantitative results with the molar amount of ammonia adsorbed are presented in Table 2.

In the case of 5%Ni-5%Zr catalysts supported on BEA zeolite, a higher reduction temperature of the catalysts affects the increase in the overall acidity, detailing a reduction in the number of weak acidic sites with an increase in the number of medium and strong acidic centers. Similarly, for the bimetallic 5%Ni-5Zr/Al_2_O_3_ catalyst, an increase in total acidity was observed with the increase in the reduction temperature of the samples. However, in this case the total acidity was caused by the increase in the amount of weak acidic centers and a slight decrease in the amount of medium and strong acidic sites for the sample after reduction at 600 °C and the increase in the medium and strong acidic centers for the sample after reduction at the highest temperature (700 °C).

For the 5%Ni-5%ZrZSM-5 zeolite catalyst, the opposite trend was observed—the measured total acidity decreased for samples reduced at higher temperatures. It is worth mentioning that for the catalyst after reduction at 500 °C, the amount of weak and medium acidic centers was dominant, for the sample reduced at 600 °C the highest amount of weak acidic sites was observed, but also an increase in strong acidic centers in comparison with the sample reduced at a lower temperature was noticed. In the case of 5%Ni-5%Zr/ZSM-5 reduced at 700 °C, it was found that the main acidic sites are medium and a significant decrease in weak and strong acidic centers was observed compared to the samples reduced at lower temperatures.

It should be mentioned that the highest amount of NH_3_ adsorbed was observed for the 5%Ni-5%ZrZSM-5 reduced at 500 °C and 5%Ni-5%ZrZSM-5 reduced at 600 °C catalysts and the smallest amount of NH_3_ adsorbed was found for 5%Ni-5%Zr/Al_2_O_3_ samples. The total acidity for the studied samples decreases in the following order:

5%Ni-5%ZrZSM-5 (500 °C) > 5%Ni-5%ZrZSM-5 (600 °C) > 5%Ni-5%ZrBEA (700 °C) > 5%Ni-5%ZrBEA (600 °C) > 5%Ni-5%ZrZSM-5 (700 °C) > 5%Ni-5%ZrBEA (500 °C) > 5%Ni-5%Zr/Al_2_O_3_ (700 °C) > 5%Ni-5%Zr/Al_2_O_3_ (600 °C) > 5%Ni-5%Zr/Al_2_O_3_ (500 °C).

In the work by Khandan et al. [48], it was observed from TPD profiles that the addition of Zr to zeolite (HM-mordenite) caused an increase in the number of moderately strong acid sites (in the temperature range of 300–500 °C) on the surface compared to the unmodified mordenite and to the system containing nickel. In addition, the Zr-HM system showed higher overall acidity than the nickel catalyst. A larger desorption peak in the temperature range of 100–300 °C was observed for the Ni-HM system than for pure zeolite. This corresponds to an increase in the number of weak acid sites. This observation can suggest that the presence of nickel and zirconium in one catalytic system can influence its acidity and allow modifying the strength of acidic centers, which could be the key factor in the catalyst’s activity and selectivity.

### 2.4. Catalytic Activity of Prepared Bimetallic Nickel-Zirconium Catalysts

The catalytic activity of prepared catalysts was performed in the hydrocracking reaction of algal oil with stable conditions of reaction: temperature: 320 °C, H_2_ pressure: 75 Bar, time: 2 h. The studied catalysts were reduced at 3 various temperatures: 500, 600 and 700 °C for 2 h in the reducing mixture of 5%H_2_-95%Ar flow before the catalytic tests. The catalytic activity results of such activated bimetallic nickel-zirconium catalysts supported on BEA zeolite, ZSM-5 zeolite and Al_2_O_3_ presented as algal oil conversion and identified fuel fraction were collected in Table 3. The highest oil conversion was observed for the hydrocracking reactions carried out with bimetallic nickel-zirconium catalysts supported on BEA zeolite and it was equal between 86–98% and this conversion decreased with the increase in the reduction temperature. In the case of bimetallic nickel-zirconium catalysts supported on ZSM-5 and Al_2_O_3_, the increase in the reduction temperature to 600 °C improved the oil conversion, but the further increase in the reduction temperature led to a significant drop in oil conversion. It seems that oil conversion could be related to the specific surface area, pore volume and average pore radius and also to the size of nickel nanoparticles and ZrO_2_ particles. The highest oil conversion was noted for the catalysts with the highest specific surface area and representing the highest total pore volume and average pore radius, which can favor breaking the long carbon chains in free fatty acids and can also be proper for large molecules of these acids present in algal oil. Moreover, for the 5%Ni-5%ZrBEA catalysts the smallest size of metallic nickel nanoparticles and the smallest size of ZrO_2_ particles were observed, which can suggest that the highest specific surface area favors the small size of metal particles and leads to better dispersion of active centers. It could also be concluded that in such catalytic systems the number of active centers is the highest and the reduction process is more effective. In the case of 5%Ni-5%ZrAl2O3 catalysts, the oil conversion is the lowest and the defined specific surface area is the smallest. It should also be noted that the size of nickel and ZrO_2_ particles was impossible to measure, which can mean that the amount of particles was very small and the reduction process was not effective.

The identified hydrocarbons were divided into groups of different kinds of fuel and selectivity to these types of fuel was presented in Table 3.

The division of the kinds of fuel usually varies in different data. In our work this distribution of products of the hydrocracking reaction was based on the research article written by M. Al-Muttaqii and co-workers published in Bulletin of Chemical Reaction Engineering and Catalysis [49]. This division establishes four main groups of hydrocarbons: gasoline, where products with carbon numbers in chains from 5 to 9 were collected; kerosene, which consisted of products with carbon numbers in the chain from 10 to 13; gasoil—the group of hydrocarbons with carbon numbers in the chain from 14 to 22; and the residue with carbon numbers higher than 22. In the case of 5%Ni-5%ZrBEA zeolite catalysts, the selectivity towards the gasoil fraction was the highest. The gasoline fraction was really small and was equal to not more than 0.39%. The kerosene fraction was not dominant, but it should be mentioned that the selectivity to these three fractions of fuel increased with the increasing activation temperature in a 5%H_2_-95%Ar flow. Despite the highest oil conversion found for these samples, the selectivity towards gasoline and kerosene was smaller than that noted for the catalysts supported on ZSM-5 zeolite and Al_2_O_3_. The selectivity towards hydrocarbons with carbon numbers in the chain higher than 22 was also the highest for the catalysts supported on BEA zeolite. This can suggest that the highest pore volume and average pore radius in these materials may be responsible for the worse cracking possibilities and the same longer chain hydrocarbon formation during the hydrocracking process of algal oil.

For the bimetallic nickel-zirconium catalysts supported on ZSM-5 zeolites, which represent the smaller pore volume and average pore radius, an increase in the selectivity towards gasoline and kerosene in comparison with 5%Ni-5%ZrBEA zeolite catalysts was observed. Also, a very similar level of selectivity towards gasoil to that found for BEA zeolite catalysts and a decrease in the selectivity towards residue compared to that found for 5%Ni-5%ZrBEA zeolite samples were observed. It is worth noting that the increase in the reduction temperature to 600 °C improved the selectivity towards gasoline and kerosene, but a further increase in the reduction temperature led to a reduction in selectivity. A similar effect was observed in the case of selectivity towards kerosene for 5%Ni-5%Zr/Al_2_O_3_ catalysts. This could be related to the sintering of metallic nickel nanoparticles and the formation of these nanoparticles with a bigger size. However, the selectivity towards gasoline remained the same for all reduction temperatures in the case of catalysts supported on alumina oxide. The selectivity towards gasoil observed for these samples increased with the increasing reduction temperature. For these bimetallic nickel-zirconium catalysts, the observed average pore radius was similar to that of the catalysts supported on ZSM-5 zeolite, but the pore volume was two times higher. It should be stressed that the selectivity towards gasoline and kerosene noted for 5%Ni-5%Zr/Al_2_O_3_ samples was the highest among all the studied bimetallic catalysts. This can suggest that the formation of hydrocarbons from the gasoline and kerosene fractions takes place on nickel nanoparticles and is not related to the shape of the support.

## 3. Discussion

The carried out research on the catalytic performance of bimetallic nickel-zirconium catalysts supported on various zeolites and alumina oxides in the hydrocracking process of algal oil suggests that their catalytic activity strictly depends on their physicochemical properties. The oil conversion is related to the specific surface area, pore volume, average pore radius and the reducibility of the studied catalysts. The key factor could also be the size of nickel and zirconium oxide nanoparticles. The highest oil conversion was observed for 5%Ni-5%Zr supported on BEA zeolite samples, for which the highest specific surface area, pore volume and average pore radius were also observed. This could be connected with the easier cracking process of the long chain of free fatty acids and also with the better shape selectivity for larger molecules. For the same reason, probably the selectivity towards the residue fraction and gasoil fraction was observed for the abovementioned catalysts. The smallest oil conversion was noted for the 5%Ni-5%Zr/Al_2_O_3_ catalysts, for which the observed reduction profile indicates the strongest interaction of nickel ions with the support and the same worse reducibility and necessity of using a higher reduction temperature. For the catalysts supported on alumina oxide, the highest selectivity towards gasoline and kerosene and the smallest selectivity towards gasoil were observed in comparison with the catalysts supported on zeolites. This may indicate that acidity is also the key factor influencing the kind of product formation in the hydrocracking of algal oil. It suggests that the smaller acidity of catalysts can favor the formation of lighter hydrocarbons and be better for gasoline and kerosene fraction production. The studies show that the use of various kinds of catalysts and the application of different support materials make it possible to control the hydrocracking process of algal oil and influence the kind of product formation and thus to control different ways of one process. The reduction temperature of prepared catalysts is one of the key factors responsible for the increase in oil conversion and selectivity to gasoline and kerosene fractions. It should be mentioned that this increase was observed for all the studied samples and was noted for an increase in reduction temperature to 600 °C in the case of 5%Ni-5%ZrZSM-5 zeolite and 5%Ni-5%Zr/Al_2_O_3_ catalysts. In the case of 5%Ni-5%ZrBEA zeolite catalysts, the increase in selectivity to lighter hydrocarbons (gasoline and kerosene fractions) grew with the increase in reduction temperature from 500 to 700 °C. However, for all the studied samples reduced at 700 °C, a decrease in oil conversion was observed. This can be connected to the increase in size of nickel nanoparticles caused by the sintering of metal particles during reduction at higher temperatures. It seems that the best temperature of activation is the reduction of prepared catalysts at 600 °C because in the case of all the catalyst samples, an increase in oil conversion and selectivity to gasoline and kerosene fractions was observed. Moreover, it should be noted that the selection of a higher reduction temperature favors an increase in selectivity to gasoil, which can mean that the choice of the reduction temperature of the studied catalysts allows controlling the hydrocracking process to produce a given kind of fuel fraction.

## 4. Materials and Methods

### 4.1. Sample Preparation

The supported bimetallic nickel-zirconium catalysts on BEA zeolite (Si/Al = 23), ZSM-5 zeolite (Si/Al = 25), and aluminum oxide were prepared by the wet co-impregnation method. The ammonium forms of BEA and ZSM-5 zeolites were purchased from Zeolyst International (Kansas City, MO, USA). Before the impregnation process, the ammonium forms of zeolites were calcined in a muffle furnace in the air atmosphere at 500 °C for 20 h to obtain the proton forms of zeolites. Then the aqueous solution of the active phase precursors was prepared from nickel nitrate hexahydrated (Ni(NO_3_)_2_∙6 H_2_O; Sigma-Aldrich, Poznan, Poland, purity 99.0%) and hydrated zirconium (IV) oxynitrate (ZrO(NO_3_)_2_∙13 H_2_O; Sigma-Aldrich, purity 99.0%). The masses of nickel and zirconium salts were prepared to correspond to 5 wt. % of metals—nickel and zirconium—in the final catalyst. The three chosen supports were impregnated with the thus-obtained aqueous solution of metal precursors for 24 h. After impregnation, the water was evaporated, and all the samples were dried at 120 °C for 2 h. Finally, the resulting solid samples were calcined at 600 °C for 4 h in the air atmosphere in a muffle furnace. For the reference sample—5%Ni-5%Zr/Al_2_O_3_ the ICP-OES analysis was performed, and the results show smaller contents of metals in this catalyst—3 and 2.5 wt. % for Ni and Zr, respectively. In this manuscript, the sample notations are original, with the given theoretical contents of metals.

### 4.2. Characterization of the Physicochemical Properties of Prepared Catalysts

The textural properties of the Ni-Zr-supported catalyst samples were determined by N_2_ adsorption at 77 K using the Brunauer–Emmett–Teller method. The BET surface area, pore volume, average pore size with the determined micropore area, external surface area and micropore volume were defined by the BET method and Dubinin–Radushkevich, BJH and t-plot calculations.

The morphology and distribution of the elements on the catalyst surfaces were studied using a S-4700 scanning electron microscope from HITACHI S-4700 equipped with an EDX detector from ThermoNoran. The sample was placed on double-sided adhesive tape and fixed to an SEM table and then sputter coated with carbon. The microscope was working at room temperature with an accelerating voltage of 25 kV and magnification of 500–5000×.

The reducibility of the prepared catalytic systems was defined by the temperature-programmed reduction technique (TPR-H_2_) using an automated AMI-1 instrument (Altamira Instruments, Pittsburgh, PA, USA). Measurements were carried out in the temperature range of 35–900 °C, while hydrogen consumption was monitored using a thermal conductivity detector (TCD).

The X-ray diffraction technique was used to determine the phase composition and the reduction behavior of the obtained zeolite catalytic materials. A PANalytical X’Pert Pro MPD diffractometer (Malvern Panalytical Ltd., Royston, UK) was used in Bragg-Brentano reflection geometry using Cu Kα radiation (k = 154.05 pm) from a sealed tube in the 2Θ angle range of 5°–90°. In addition, the PANalytical HighScore Plus software package (ver. 4.9) was used for phase analysis in conjunction with the International Center for Diffraction Data (ICDD PDF-2 ver. 2020) powder diffraction database of standard reference materials. The “in situ” measurements of the reduction process were carried out in a chamber in a gaseous mixture flow of 5%H_2_-95%Ar in the temperature range of 500–900 °C with measurements recorded every 50 °C starting from 500 °C. The determination of Ni and ZrO_2_ particle sizes was performed using the Scherrer equation.

The acidic properties of the synthesized catalytic materials were studied by the temperature-programmed desorption (TPD) technique using ammonia as a probe molecule. TPD-NH_3_ measurements were carried out in a quartz microreactor. Prior to each experiment, catalyst samples were reduced “in situ” at 500 °C, 600 °C, or 700 °C for 2 h in a 5%H_2_-95%Ar reduction mixture flow; the flow rate was 40 mL/min. Then, ammonia was adsorbed on the surface of the catalyst sample at 100 °C for 15 min, after which the system was flushed with helium for 15 min at 100 °C to remove physically adsorbed NH_3_ from the catalyst surface. TPD-NH_3_ measurements were carried out in the temperature range of 100–600 °C at a constant helium flow rate of 40 mL/min. Ammonia desorption from the acidic sites of the catalysts was identified using a TCD detector.

### 4.3. Catalytic Activity Performance and Chromatographic Analysis of Products of Hydrocracking of Alagal Oil

Hydrocracking with Ni-Zr zeolite catalysts was carried out in an autoclave (manufactured by Parr, Moline, IL, USA; reactor volume 50 mL) equipped with a mechanical mixing device. The substrate was algal oil of the Spirulina Platensis species. The process was carried out for 2 h at 320 °C with a hydrogen pressure of 75 bar. In all catalytic tests, the amount of catalyst used was about 1% by weight of the oil. In order to activate the system, the catalysts were subjected to a reduction process in a reducing mixture of 5%H_2_-95%Ar at a flow rate of 50 cm^3^/min for 2 h. The catalysts used for the experiments were reduced at different reduction temperatures of 500 °C, 600 °C and 700 °C to check their impact on catalytic activity.

#### Chromatographic Analysis of Obtained Products and Defining of Oil Conversion

The obtained hydrocracking reaction products were analyzed by chromatographic methods. Triglyceride conversion was determined using a high-performance liquid chromatograph (Shimadzu), equipped with a C-18 column (5 um, 4.6 × 250) and a DAD detector (wavelength: λ = 205 nm). The eluent was a mixture of 2-isopropanol-hexane (4/5) and methanol. A sample of 100 μL was diluted with 900 μL of n-hexane. All chemicals were purchased from CHEMPUR, Piekary Slaskie, Poland. The mobile phase gradient used during each experiment is shown in Table 4.

The composition of the resulting hydrocracking products was determined by gas chromatography coupled to mass spectrometry (GC-MS) using a Zebron capillary column, model ZB-5MSplus, 30 m in length, I.D. 0.25 mm and film thickness 0.25 μm. The conditions for the whole analysis are given in Table 5, Table 6 and Table 7. As for HPLC analyses, 100 μL was taken from the sample of final products and diluted with 900 μL of n-hexane.

## 5. Conclusions

In the presented work, the catalytic activity of bimetallic nickel-zirconium catalysts supported on different porous materials in the hydrocracking process of algal oil was described. It was proved that the kind of support can significantly affect the physicochemical and catalytic properties of catalysts.

⮚ The acidity of the studied catalysts decreased in the following order:

5%Ni-5%ZrZSM-5 (500 °C) > 5%Ni-5%ZrZSM-5 (600 °C) > 5%Ni-5%ZrBEA (700 °C) > 5%Ni-5%ZrBEA (600 °C) > 5%Ni-5%ZrZSM-5 (700 °C) > 5%Ni-5%ZrBEA (500 °C) > 5%Ni-5%Zr/Al_2_O_3_ (700 °C) > 5%Ni-5%Zr/Al_2_O_3_ (600 °C) > 5%Ni-5%Zr/Al_2_O_3_ (500 °C).

⮚ The worst reducibility and the stronger metal-support interaction were observed for 5%Ni-5%Zr/Al_2_O_3_ catalyst;
⮚ The highest conversion and selectivity towards the gasoil fraction were noted for 5%Ni-5%ZrBEA zeolite catalyst;
⮚ The highest selectivity towards the gasoline and kerosene fractions was found for 5%Ni-5%Zr/Al_2_O_3_ catalysts;
⮚ The catalytic activity suggests that the specific surface area, pore volume, average pore radius, reduction behavior and acidity are the key factors influencing the course of the hydrocracking of algal oil process. The application of different porous materials as the support for catalysts for this reaction can allow controlling the kind of product formation.

## Figures and Tables

**Table 1:** Textural properties of bimetallic nickel-zirconium catalysts supported on BEA zeolite, ZSM-5 zeolite and Al_2_O_3_ after their calcination at 600 °C in air and reduction at 500, 600 and 700 °C for 2 h in 5%H_2_-95%He gaseous mixture flow.

Catalyst	Specific Surface Area (BET)	Micropore Area	External Surface	Total Pore Volume (BJH)	Micropore Volume	Average Pore Radius (BJH)
	(m^2^/g)	(m^2^/g)	(m^2^/g)	(cm^3^/g)	(cm^3^/g)	(nm)
**HBEA (Si/Al = 23)**	526	331	195	0.74	0.17	7.1
**5%Ni-5%ZrBEA (red. 500 °C)**	449	281	167	0.63	0.13	7.2
**5%Ni-5%ZrBEA (red. 600 °C)**	449	272	177	0.62	0.14	6.7
**5%Ni-5%ZrBEA (red. 700 °C)**	445	266	179	0.63	0.14	6.7
**HZSM-5 (Si/Al = 25)**	281	257	24	0.11	0.13	2.4
**5%Ni-5%ZrZSM-5 (red. 500 °C)**	277	212	65	0.06	0.11	2.1
**5%Ni-5%ZrZSM-5 (red. 600 °C)**	271	208	64	0.07	0.11	2.4
**5%Ni-5%ZrZSM-5 (red. 700 °C)**	269	208	62	0.07	0.11	2.4
**5%Ni-5%Zr/Al_2_O_3_ (red. 500 °C)**	115	-	-	0.21	-	2.5
**5%Ni-5%Zr/Al_2_O_3_ (red. 600 °C)**	112	-	-	0.21	-	2.6
**5%Ni-5%Zr/Al_2_O_3_ (red. 700 °C)**	111	-	-	0.21	-	2.6

**Table 2:** The amount of chemically adsorbed NH_3_ calculated from TPD-NH_3_ data obtained for 5%Ni-5%ZrBEA zeolite, 5%Ni-5%ZrZSM-5 zeolite and 5%Ni-5%Zr/Al_2_O_3_ after calcination at 600 °C for 4 h and reduction at various temperatures: 500, 600 and 700 °C for 2 h.

Catalytic Systems	Total Acidity (mmol/g) 100–600 °C	Distribution of Acid Sites		
		Weak (mmol/g) 100–300 °C	Moderate (mmol/g) 300–500 °C	Strong (mmol/g) 500–600 °C
**5%Ni-5%Zr/BEA (red. 500 °C)**	2.15	1.08	0.74	0.32
**5%Ni-5%Zr/BEA (red. 600 °C)**	2.58	0.97	1.06	0.56
**5%Ni-5%Zr/BEA (red. 700 °C)**	2.71	0.87	1.13	0.72
**5%Ni-5%Zr/ZSM-5 (red. 500 °C)**	3.06	1.14	1.19	0.73
**5%Ni-5%Zr/ZSM-5 (red. 600 °C)**	2.89	1.10	0.91	0.88
**5%Ni-5%Zr/ZSM-5 (red. 700 °C)**	2.26	0.66	1.11	0.49
**5%Ni-5%Zr/Al_2_O_3_ (red. 500 °C)**	1.34	0.46	0.54	0.34
**5%Ni-5%Zr/Al_2_O_3_ (red. 600 °C)**	1.57	0.77	0.49	0.30
**5%Ni-5%Zr/Al_2_O_3_ (red. 700 °C)**	1.66	0.40	0.66	0.60

**Table 3:** The algal oil conversion and selectivity towards various fractions of fuel observed for bimetallic nickel-zirconium catalysts supported on BEA zeolite, ZSM-5 zeolite, or Al_2_O_3_, measured by chromatographic data.

Sample	Red. 500 °C	Red. 600 °C	Red. 700 °C
	Conversion of oil (%)		
5%Ni-5%ZrBEA	98	94	86
5%Ni-5%ZrZSM-5	82	86	73
5%Ni-5%ZrAl_2_O_3_	57	68	51
	Gasoline (<C_10_) (%)		
5%Ni-5%ZrBEA	0.09	0.33	0.39
5%Ni-5%ZrZSM-5	4.73	7.10	2.05
5%Ni-5%ZrAl_2_O_3_	7.70	7.63	7.50
	Kerosene (C_10_–C_13_) (%)		
5%Ni-5%ZrBEA	6.35	8.83	9.82
5%Ni-5%ZrZSM-5	6.55	16.27	12.36
5%Ni-5%ZrAl_2_O_3_	20.31	22.54	11.95
	Gasoil (C_14_–C_22_) (%)		
5%Ni-5%ZrBEA	76.79	89.71	78.16
5%Ni-5%ZrZSM-5	81.73	71.04	79.46
5%Ni-5%ZrAl_2_O_3_	60.28	66.90	71.08
	Residue (C > 22) (%)		
5%Ni-5%ZrBEA	16.77	1.13	11.73
5%Ni-5%ZrZSM-5	7.00	5.60	6.14
5%Ni-5%ZrAl_2_O_3_	11.71	2.93	9.48
*The crystallite sizes of Ni particles and ZrO_2_ calculated from the XRD data using* *the Scherrer equation*			
	***500* °C** ***Ni/ZrO*_2_** **(nm)**	***600* °C** ***Ni/ZrO*_2_** **(nm)**	***700* °C** ***Ni/ZrO*_2_** **(nm)**
5%Ni-5%ZrBEA	***6/5***	***8/5***	***13/6***
5%Ni-5%ZrZSM-5	***13/9***	***14/10***	***19/11***
5%Ni-5%ZrAl_2_O_3_	***-***	***-***	***9/5***

**Table 4:** Phase gradient used in the HPLC measurements.

Mobile Phase Gradient			Flow Rate (mL/min)
Time (min)	Solvent A (%)	Solvent B (%)	
0	100	0	0.9
20	100	0	0.9
45	0	100	0.9
70	0	100	0.9
75	100	0	0.9

Solvent A: Methanol; Solvent B: 2-Propanol/Hexane = 4/5; Injection Volume: 1 μL; Column Temperature: 25 °C.

**Table 5:** Analysis settings of the gas chromatography apparatus GC-2010.

Column Oven Temperature	35.0 °C
Injection Temperature	320.00 °C
Injection Mode	Split
Injection Volume	1.00 μL
Flow Control Mode	Linear Velocity
Pressure	22.9 kPa
Total Flow	10.7 mL/min
Column Flow	0.70 mL/min
Linear Velocity	30.0 cm/s
Purge Flow	3.0 mL/min
Split Ratio	10.0

**Table 6:** Settings for the oven temperature program.

Rate (°C/min)	Temperature (°C)	Hold Time (min)
-	35.0	5.00
15.00	320.0	6.00

**Table 7:** Analysis settings of the mass spectrometer (GCMS-QP2010 SE) coupled to the gas chromatography apparatus GC-2010.

Ion Source Temperature	220.00 °C
Interface Temperature	280.00 °C
Solvent Cut Time	2.50 min
Detector Gain Mode	Relative to the Tuning Result
Detector Gain	0.74 kV + 0.00 kV
Threshold	0
Start Time	2.70 min
End Time	30.00 min
ACQ Mode	Scan
Event Time	0.30 s
Scan Speed	1666
Start *m*/*z*	35.00
End *m*/*z*	500.00
Sample Inlet Unit	GC

## Data Availability

Data are available on the request.
